# The effectiveness of antenatal care programmes to reduce infant mortality and preterm birth in socially disadvantaged and vulnerable women in high-income countries: a systematic review

**DOI:** 10.1186/1471-2393-11-13

**Published:** 2011-02-11

**Authors:** Jennifer Hollowell, Laura Oakley, Jennifer J Kurinczuk, Peter Brocklehurst, Ron Gray

**Affiliations:** 1 National Perinatal Epidemiology Unit, University of Oxford, Old Road Campus, Oxford, OX3 7LF, UK

## Abstract

**Background:**

Infant mortality has shown a steady decline in recent years but a marked socioeconomic gradient persists. Antenatal care is generally thought to be an effective method of improving pregnancy outcomes, but the effectiveness of specific antenatal care programmes as a means of reducing infant mortality in socioeconomically disadvantaged and vulnerable groups of women has not been rigorously evaluated.

**Methods:**

We conducted a systematic review, focusing on evidence from high income countries, to evaluate the effectiveness of alternative models of organising or delivering antenatal care to disadvantaged and vulnerable groups of women vs. standard antenatal care. We searched Medline, Embase, Cinahl, PsychINFO, HMIC, CENTRAL, DARE, MIDIRS and a number of online resources to identify relevant randomised and observational studies. We assessed effects on infant mortality and its major medical causes (preterm birth, congenital anomalies and sudden infant death syndrome (SIDS))

**Results:**

We identified 36 distinct eligible studies covering a wide range of interventions, including group antenatal care, clinic-based augmented care, teenage clinics, prenatal substance abuse programmes, home visiting programmes, maternal care coordination and nutritional programmes. Fifteen studies had adequate internal validity: of these, only one was considered to demonstrate a beneficial effect on an outcome of interest. Six interventions were considered 'promising'.

**Conclusions:**

There was insufficient evidence of adequate quality to recommend routine implementation of any of the programmes as a means of reducing infant mortality in disadvantaged/vulnerable women. Several interventions merit further more rigorous evaluation.

## Background

In recent years, infant mortality in most parts of the world has shown a steady decline [1]. Across high-income OECD countries as a whole, the average infant mortality rate declined from 12.2 deaths per 1000 live births in 1980 to 4.9 deaths per 1000 live births in 2008; and in the United Kingdom the rate showed a similar decline, from 12.1 deaths per 1000 live births in 1980 to 4.9 deaths per 1000 live births in 2008. But throughout this period infant mortality has shown marked and persistent socioeconomic gradients within countries, even in countries with universal healthcare access [2-4]. Immaturity related conditions and congenital anomalies are the two main causes of infant deaths in high-income countries [5-7]; and for both of these causes mortality rates exhibit socioeconomic gradients with the highest rates occurring in the most socioeconomically disadvantaged groups [8,9]. A number of so-called vulnerable groups also suffer disproportionately high rates of infant mortality (and other adverse perinatal outcomes), or have a high prevalence of risk factors for poor pregnancy outcome/infant health: such groups include teenagers[5,10], many black and minority ethnic groups [10,11], homeless women [12,13], prisoners [12,14], women who have experienced domestic violence [15], asylum seekers and refugees [12], women with mental illness [16] and women with substance abuse problems [12,17,18].

A review of the international effectiveness literature conducted at the NPEU in 2008 (updated in 2009 [19]) confirmed the paucity of relevant systematic review level evidence relating to infant mortality and related outcomes in disadvantaged populations; and a review of UK interventions to improve perinatal outcomes in disadvantaged groups found limited UK evidence of effective interventions for disadvantaged childbearing women [12].

Antenatal care is generally thought to be an effective method of improving outcomes in pregnant women and their babies, although many antenatal care practices have not been subject to rigorous evaluation [20]. One review from the early 1990 s evaluated 'prenatal care packages' [21] but found only five studies of adequate quality which evaluated the effect of the programme on gestational age at birth and/or infant mortality, two of which (Nurse Home Visitation [22]; and case management [23]) were found to have a positive effect on the relevant outcome measure.

Other systematic reviews have evaluated the effect of specific antenatal care packages on preterm birth (PTB) and infant mortality, including: alternative ways of delivering antenatal care to Australian indigenous women [24]; telephone support and home visiting programmes [25,26]; continuity of caregiver during pregnancy and childbirth [27,28]; and modified timing and frequency of antenatal care visits [29-31].

These reviews found that telephone support [25], home visits/social support [25,26] and continuity of care [27,28] had beneficial effects on a range of measures of maternal and infant health and wellbeing, but none of these interventions was found to have a statistically significant effect on infant mortality or PTB. One review [24] found some studies that reported beneficial effects of some interventions targeting Australian indigenous women, but the authors of the review concluded that the evidence was flawed.

In the light of the paucity of up to date evidence relating to the effectiveness of antenatal care programmes as a means of reducing infant mortality in disadvantaged groups of women, the aim of this systematic review was to identify the best available evidence on the effectiveness of interventions focused on the delivery and organisation of antenatal care to reduce infant mortality, or one of its three major causes (PTB, congenital anomalies, sudden infant death syndrome/sudden unexpected death in infancy (SIDS/SUDI)) in socially disadvantaged and vulnerable groups of women and other specific groups, such as teenagers and substance abusers, with risk factors for adverse birth outcomes strongly associated with social disadvantage.

## Methods

Because the review findings were aimed at policy makers and healthcare managers, our approach incorporated some of the iterative methods of interpretive synthesis proposed by Lomas and others for policy research synthesis[32]: the research question, PICO criteria and methods for the identification and screening of studies were pre-specified but decisions regarding how best to analyse and present findings from the included studies were taken iteratively by the authors in the light of the available material.

### Criteria for including studies in the review

Criteria for including studies in the review are summarised in Table 1.

**Table 1:** Criteria for including studies in the review

	Inclusion criteria
Study design	Experimental or observational effectiveness evaluation, with control or comparator group
Population	Socially disadvantaged or vulnerable populations*
	Other specified at risk population: teenagers, obese pregnant women, substance users, alcohol misusers, women who are HIV positive
Intervention	Intervention involving the organisation and/or delivery of:
	• comprehensive antenatal care
	• components of antenatal care provided in the context of normal antenatal care
	and/or
	• Stand alone interventions involving the provision of health or social care to pregnant women delivered as *an adjunct to *normal antenatal care
	Exclusions:
	• stand-alone interventions targeting pregnant women not delivered and/or evaluated in conjunction with standard antenatal care
	• clinical interventions, unless evaluated in the context of a broader package of antenatal care
	• interventions with a focus on labour/birth or the periconceptional period
	• interventions involving only opiate substitution
Comparator	Standard antenatal care or a specified alternative model of antenatal care
Outcome	• Preterm birth (or "preterm labour") expressed as the number/proportion of women delivering before 37 weeks gestation (or some other cut-off point <37 weeks)
	• Any measure of neonatal/infant mortality, but excluding perinatal mortality
	• Birth prevalence of congenital anomalies
	• SIDS/SUDI
Type of publication	Journal articles reporting primary research in English and non-English language journal articles with an English Language abstract
Geographical area	OECD member countries, excluding Mexico and Turkey**
Time period	Published 1990 onwards

*Including: women living in deprived areas, disadvantaged minority ethnic/racial groups, women in prison, travellers, homeless women, asylum seekers and refugees, recently arrived migrants/other immigrant groups, victims of abuse, women with mental illness/mental health problems, women with learning disabilities, sex workers.

**High-income countries with low infant mortality.

### Methods for identification of studies

We searched the following databases in mid-August 2008 for reports of primary research studies published between January 1990 and July 2008: Medline, Embase, Cinahl, PsycINFO, HMIC, CENTRAL, Database of Abstracts of Reviews of Effects (DARE), MIDIRS. We used a search strategy which combined MeSH terms/keyword and text search terms relating to the outcomes, interventions and populations of interest (See additional file 1).

We additionally searched a number of other specialist databases, including the Cochrane Database of Systematic Reviews, and online resources (see additional file 1 for list) to identify potentially eligible primary reports and also review articles, guidelines, and other reports that might contain relevant citations. The bibliographies of the latter were inspected to identify relevant primary reports.

Two reviewers independently assessed titles/abstracts of all potentially relevant/eligible references using a simple checklist of exclusion criteria. The full-text of articles not excluded on title/abstract was screened independently by two reviewers using a more detailed checklist of exclusion and inclusion criteria. At both stages, discrepancies were discussed and the opinion of a third reviewer sought where necessary to reach a final decision regarding eligibility.

### Quality assessment

Internal validity was assessed using the 'Graphical appraisal tool for epidemiological studies' (GATE) developed by Jackson and colleagues [33]. GATE is a generic quality appraisal tool which can be applied to a wide range of experimental and observational study designs [34] and thus avoided the need to use different tools according to the study design.

Randomised studies were assessed by a single reviewer; observational studies were assessed independently by two reviewers. Each reviewer completed the checklist and assigned an overall assessment of internal validity according to the GATE criteria. Where the two assessments (observational studies only) differed, a third reviewer re-assessed the studies and a final rating was assigned following review and discussion of the three independently completed checklists. Risk of bias was assessed at the outcome level (PTB or infant mortality); where both PTB and infant mortality were reported, the assessment was based on the outcome considered to be the 'primary outcome'.

Prior to undertaking the study GATE assessments, reviewers completed and discussed a minimum of five 'training assessments' to ensure that the tool was being correctly and consistently applied.

### Data extraction

A data extraction and coding form was developed and loaded into specialist review software (Eppi-Reviewer[35]). Descriptive data were extracted and entered by one reviewer and checked by a second reviewer. Outcome data were extracted and coded/entered independently by two reviewers and checked for agreement.

### Assessment of evidence of effectiveness

Two reviewers independently assessed and coded the authors' conclusions regarding the effect of the intervention on the outcomes of interest. For each of the outcomes reported, conclusions were coded as follows: (a) statistically significant effect on the outcome; (b) effect consistent with a beneficial effect but effect not statistically significant and/or cautious interpretation of findings recommended; (c) no evidence of beneficial effect; (d) no conclusion stated.

For studies having 'adequate' internal validity ('good' or 'mixed' GATE quality assessment), the reviewers also independently assessed and coded the evidence of effectiveness for individual outcomes, taking into account the strength and limitations noted in the GATE checklist. Evidence of effectiveness was coded as follows: (a) study demonstrates a beneficial effect on the outcome; (b) study inconclusive but suggestive of a beneficial effect; (c) study does not provide convincing evidence of a beneficial effect.

Discrepancies in coding were resolved by discussion with a third reviewer.

## Results

### Studies included

**Figure 1:**
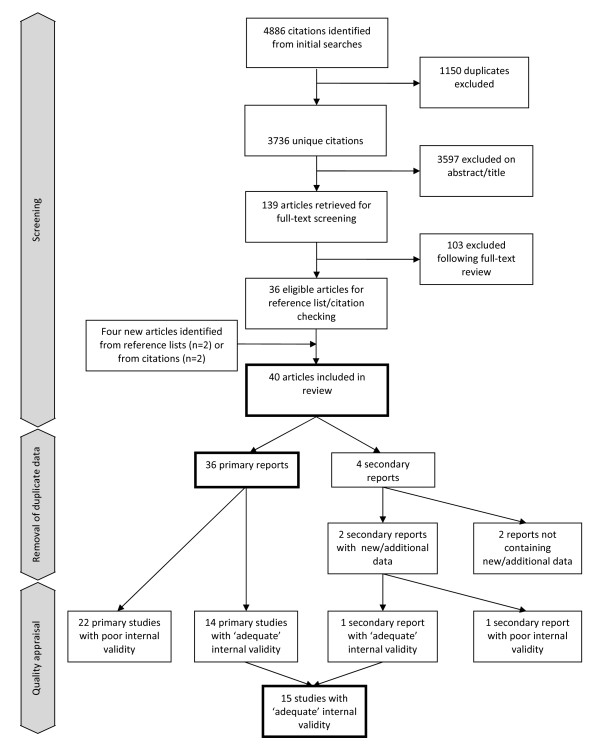
**Screening and study inclusion flow chart**.

Our initial searches identified 3736 unique citations. Of these, 3597 were excluded on title/abstract alone and a further 103 were excluded following full-text review. (See additional file 2 for reasons for exclusion.) Four new articles were identified from reference lists and citations. In total, 40 eligible articles were included (see Figure 1) relating to 36 distinct interventions and/or studies. Two of the four 'secondary' reports, did not provide additional relevant data [36,37] (and are not considered further); and two provided additional data supplementing those provided in the 'primary' reports (one reported additional data on neonatal mortality [38] and one reported effectiveness data for a subgroup of interest [39]).

The characteristics of the 36 included primary studies are shown in Table 2.

**Table 2:** Characteristics of the included primary studies

	Number (%) of studies
**Year of publication**	
1990-1994	8 (22)
1995-1999	9 (25)
2000-2004	15 (42)
2005-2008 (part year)	4 (11)
	
**Country**	
USA	26 (72)
Australia	4 (11)
U.K.	4 (11)
Canada	1 (3)
Greece	1 (3)
	
**Study design**	
RCT-individually randomized	7 (19)
RCT-cluster randomized	2 (6)
Retrospective cohort study	12 (33)
Prospective cohort study	6 (17)
Cohort study (unspecified)	2 (6)
Mixed retrospective/prospective cohort study	1 (3)
Before and after study	6 (17)
	
Outcomes reported*	
PTB/preterm labour	32 (89)
Infant mortality	5 (14)
Neonatal mortality	6 (17)
Congenital anomalies	6 (17)

* Not mutually exclusive

### Outcomes evaluated

All included studies reported PTB/preterm labour and/or a measure of neonatal/infant mortality as an outcome (Table 2). Six studies [40-45] additionally reported congenital anomalies: this outcome is not considered further in this review because the low event rate, small combined sample size across studies and diversity of interventions meant that no conclusions could be drawn regarding intervention effects on this outcome. None of the included studies evaluated effects on SIDS/SUDI.

### Quality of evidence

Eight of the nine included randomised controlled trials (RCTs) were assessed as having 'adequate' ('good' or 'mixed') internal validity, and one was rated 'poor'. Of the 27 primary observational studies, six were assessed as having 'adequate' internal validity (none 'good'; 6 'mixed') and 21 as 'poor' (See additional file 3).

Overall, fifteen of the studies (14 primary studies [23,41-43,46-55] and one secondary report providing supplementary data[38]) were considered to have 'adequate' internal validity.

### Interventions

Twenty studies related to interventions targeting and/or evaluated in socioeconomically disadvantaged/deprived populations of which eight were aimed specifically at disadvantaged women with additional clinical risk factors for PTB or LBW. Seventeen of these studies were conducted in the USA, with most targeting medically indigent and/or Medicaid eligible women.

The other sixteen primary studies related to interventions targeting or evaluated in specific vulnerable population: nine targeted pregnant teenagers, four targeted pregnant substance users, two targeted pregnant indigenous Australians, and one intervention targeted pregnant women who were HIV positive. One further secondary report [38] provided data on the effectiveness of the latter intervention in a sub-group of substance using, HIV positive women.

Twenty-three of the studies evaluated alternative of models of delivering comprehensive antenatal care and 13 evaluated interventions provided as an adjunct to comprehensive antenatal care, including home visiting, nutritional programmes, case management/care coordination and substance abuse programmes provided alongside standard antenatal care. An overview of the intervention characteristics by target population is given in additional file 4. A more detailed description of the interventions is available elsewhere [19].

The fifteen studies assessed as having adequate internal validity are described in Tables 3 and 4.

**Table 3:** Studies evaluating comprehensive antenatal care programmes

Study/ Country	Setting	Target population	Study design	Intervention
**a) Programmes targeting socioeconomically disadvantaged women without specific clinical risk factors for PTB/LBW**				
				
***Group antenatal care***				
Ickovics, 2003/ USA	Three public antenatal clinics in Atlanta, Georgia and New Haven, serving predominantly low-income, uninsured (Medicaid or self- pay) minority women.	Women without severe medical or psychiatric problems who entered antenatal care at one the three study clinics at 24 or less weeks'gestation between August 1999 and March 2002.	Prospective cohort study	Groups of 8-10 women with similar estimated due date receive the majority of their antenatal care in a communal/group setting. Groups meet periodically (typically fortnightly) with each group led by a trained practitioner. The group care model emphasizes education, skills- building, peer support and personal empowerment.
Ickovics, 2007/ USA	Publicly funded obstetric clinics in two university affiliated hospitals in Connecticut and Georgia.	Women aged less than 25 entering antenatal care at the two study sites between September 2001 and December 2004; less than 24 weeks' gestation; no "high-risk" medical problems (e.g. HIV); consenting to randomization. Multiple gestations excluded in PTB `analysis.	Randomised controlled Trial	See above (Ickovics, 2003).
***Temple Infant and Parent Support Services (TIPPS) programme***				
Reece, 2002/ USA	Community and hospital based maternity services in North Philadelphia, Pennsylvania.	Medically indigent women who enrolled in the intensive maternity care programme(TIPPS) or who enrolled in usual antenatal care at the study hospital	Prospective cohort study	A comprehensive multidisciplinary service which includes complete antenatal and delivery care, well baby care, health education, nutritionist care and counselling and psychosocial care and a range of components to increase uptake and remove barriers to care, e.g. outreach teams interface with community-based organizations to identify pregnant women who are not receiving antenatal care.
***Tennessee Medicaid Managed Care programme (TennCare)***				
Conover, 2001/ USA	Antenatal services for Medicaid eligible women in Tennessee and North Carolina.	Women resident in the two study areas delivering a singleton live births in 1993 and 1995. Study populations NOT restricted to Medicaid eligible women	Before and after study with an adjacent US state as a control group.	A public medical assistance programme which delivers antenatal care through a 'managed care' model.
**b) Programmes providing enhanced antenatal care to socioeconomically disadvantaged women with additional clinical risk factors for PTB/LBW**				
				
***West Los Angeles Preterm Prevention Project***				
Hobel, 1994/ USA	Public antenatal clinics in West Los Angeles, California.	Women with a first antenatal clinic visit at one of the study sites between 1983 and 1986 and with a completed risk assessment indicating high-risk of PTB. Multiple pregnancies, those that aborted at <20 weeks gestation and those that resulted in stillbirth or major congenital anomaly excluded.	Cluster randomised controlled trial	Clinic-based enhanced antenatal care for high risk women. Eligible women attending the clinics providing the programme receive more frequent visits (every two weeks), pre-term prevention education (three classes covering "identification of pre-term labour, steps to take if signs or symptoms occurred, prevention strategies and what to expect at the hospital") as well as psychosocial and nutritional screening and crisis intervention.
***Alabama augmented antenatal care programme for high risk women***				
Klerman, 2001/ USA	Public health care system, Jefferson County, Alabama.	African-American, Medicaid- eligible pregnant women seeking antenatal care from the Jefferson County Department of Health between March 1994 and June 1996; women at least 16 yrs old, less than 26 weeks' gestation, with a score of 10 or higher on a risk assessment scale (medical and social factors, including prior PTB, low pre-pregnancy weight, no car for transportation). Women with alcoholism, substance abuse, asthma, cancer, diabetes, epilepsy, high blood pressure, sickle cell disease or HIV/AIDS were excluded.	Randomised controlled Trial	Higher-risk women receive augmented care at a specially created *Mother* *and Family Specialty Center*. The programme focuses on informing women about their risk conditions and about what behaviour might improve their pregnancy. The programme includes elements covering smoking cessation,weight gain and vitamin-mineral supplementation and amelioration of psychosocial stress/isolation. Other features include group sessions, regular standing appointments, evening hours where needed, appointment reminders, transportation, and on-site childcare.
**c) Programmes targeting other vulnerable/at risk groups**				
				
**New York Prenatal Care Assistance Program (PCAP)**				
Newschaffer, 1998/USA	New York State Medicaid antenatal clinics.	HIV infected, drug abusing, Medicaid claimants who delivered a singleton between January 1993 and September 1994.	Retrospective cohort Study	The programme provides enhanced antenatal care to low income women through a network of accredited hospital clinics. The clinics receive financial incentives to providers to improve basic elements of management and coordination of antenatal care. PCAP accredited clinics must: provide patient outreach to facilitate timely prenatal care; meet frequency and content of care standards set by the American College of Obstetricians and Gynaecologists; conduct comprehensive risk assessment for adverse outcomes; develop prenatal care plans; and provide nutritional services, health education, psychological assessment and HIV related services involving testing, counselling and management referrals.
Turner, 2000/USA	USA. Public antenatal care services, New York, New York State	HIV-infected, New York State Medicaid enrolled women delivering a live- born singleton infant between January 1993 and October 1995	Retrospective cohort Study	See above (Newschaffer, 1998)

**Table 4:** Studies evaluating programmes provided as an adjunct to comprehensive antenatal care

Study/ Country	Setting	Target population	Study design	Intervention
**a) Interventions aimed at socioeconomically disadvantaged women**				
				
***Home visiting***				
Kafatos, 1991/Greece	Rural primary health care clinics in Florina, a socioeconomically disadvantaged rural region in Northern Greece.	Women living in a socioeconomically disadvantaged rural area	Cluster randomised controlled trial	An outreach health education/counselling service provided by nurses attached to rural primary health clinics. Women receive regular (fortnightly) nurse home visits with an emphasis on nutritional counseling covering food sources and the methods for selecting a balanced diet; instruction in practical techniques to improve the quality of the woman's diet including selection of foods with a high nutrient value and preparation/preservation techniques to reduce the loss of nutrients). Other themes covered during pregnancy included general hygiene, preparation for delivery, breastfeeding and care of the newborn. Home visits continued after delivery until the infant was 12 months old; these visits focused on infant health topics.
Kitzman, 1997/USA	Public system of obstetric care, Memphis, Tennessee.	Predominantly African- American, low-income women with multiple socio-demographic risk factors (unmarried, unemployed and/or less than 12 years education)	Randomised controlled trial	A programme based on the 'Elmira'/Family Nurse Partnership model. The antenatal aspect of the interventions (which also includes post natal home visits) involves an average of 7 home visits focusing on improving health-related behaviour (nutrition, smoking, alcohol and illegal drug use). Women are also taught to recognize the signs and symptoms of pregnancy complications and to act appropriately if these occur; and attention is paid to compliance with treatment and to urinary tract infections (UTIs) and sexually transmitted diseases (STDs).
***Maternity care co-ordination***				
Buescher, 1991/USA	Services for Medicaid eligible women, North Carolina.	Low-income women	Retrospective cohort study	The care coordinators help Medicaid-eligible women receive services and also provide to provide social and emotional support. The programme includes outreach, to help women apply for Medicaid, assessment (psychosocial, nutritional, medical, educational and financial), service planning (development of an individualized plan and provision of assistance to access services), coordination and referral, follow up and monitoring and education and counselling.
**b) Interventions aimed at or evaluated in socioeconomically disadvantaged women with additional risk factors for PTB/LBW**				
				
***Home visiting/telephone support***				
Bryce, 1991/Australia	Three public hospital antenatal clinics in Perth and the offices of 87 obstetricians and general practitioners in western Australia.	Women with a prior PTB or other specified risk factors for adverse pregnancy outcome. Intervention not restricted to socioeconomically disadvantaged women but stratified analysis of intervention effect by social class reported	Randomised controlled trial	Higher-risk women receive home visits from midwives at roughly 4-6 weekly intervals (more frequently if requested by the woman) with intervening telephone calls. The midwives provide expressive support ("empathy, understanding, acceptance, ...") and are instructed to provide instrumental support ("information, advice and material aid") only on request. Physical antenatal care is provided only in an emergency.
Moore, 1998/USA	Public health clinic, Winston-Salem, North Carolina	Low-income African- American women and low-income white women with additional risk factors for PTB	Randomised controlled trial	Higher-risk women receive a booklet and additional instruction about the signs and symptoms of preterm labour followed by three scheduled nurse phone calls per week. Each call includes an assessment of health status ("perception of uterine contractions and other pregnancy changes, color of urine as an assessment of hydration, number of meals eaten, number of cigarettes smoked, alcohol and drug use, and ingestion of a prenatal vitamin capsule on the previous day"); recommendations based on the assessment; and a discussion of any additional issues important to the mother
Oakley 1990/UK	Four hospital antenatal clinics	Disadvantaged, predominantly 'working class' women with a prior LBW birth.	Randomised controlled trial	A structured social support intervention consisting of a minimum of three antenatal home visits at 14, 20 and 28 weeks, plus two telephone contacts. Midwives engage in a semi-structured, open ended discussion with mothers on topics of the mother's choice; the midwives provide advice or information only if requested and do not provide clinical care (but may refer a mother for care if required)
**c) Interventions evaluated in other vulnerable/at risk groups**				
				
***Higgins Nutrition Intervention Program***				
Dubois, 1997/Canada	Subjects recruited from 15 Montreal area hospitals but location/setting of the Montreal Diet Dispensary unclear.	Pregnant adolescents	Retrospective cohort study	A nutritional programme delivered by trained dieticians as an adjunct to routine antenatal care. The programme has four elements: assessment of risks for the pregnancy; determination of an individualized "dietary prescription"; teaching of food consumption patterns that meet the individual's requirements while respecting pre-existing food habits; and follow-up and supervision by the same dietician at 2-week intervals.

## Effectiveness

### Comprehensive antenatal care programmes

Eight studies of adequate quality evaluated comprehensive antenatal care programmes. Results are summarized in Table 5.

**Table 5:** Effectiveness of comprehensive antenatal care programmes

Study	Study groups/sample size	Effectiveness		Evidence of effectiveness Authors' conclusion/reviewer assessment	
		
		PTB outcome	Neonatal/infant mortality outcome	PTB	Neonatal/ infant mortality
**a) Programmes targeting socioeconomically disadvantaged women without specific clinical risk factors for PTB/LBW**					
					
***Group antenatal care***					
Ickovics, 2003	229 antenatal care attendees who volunteered to receive group antenatal care vs. 229 antenatal care attendees selected from the women who did not volunteer to receive group antenatal care, matched on age, race/ ethnicity, parity and date of delivery.	Unadjusted % PTB (<37 weeks): 9.2% vs. 9.6%, p = 0.83. Unadjusted % early PTB (<33 weeks): 0.9% vs. 3.1% Unadjusted % late PTB (33-36.9 weeks): 8.3% vs. 6.5%	*Neonatal deaths, n (%)*: 0 (0%) vs. 3 (1.3%)	Possibly/No	No/No
Ickovics, 2007	625 women randomised to group antenatal care vs. 370 women randomised to individual antenatal care.	Adjusted % PTB (<37 weeks): 9.8% vs. 13.8%, p = .045 Adjusted odds ratio (95% CI) for PTB: 0.67 (0.44-0.98)	N/A	Yes/Yes	N/A
***Temple Infant and Parent Support Services (TIPPS) programme***					
Reece, 2002	380 women enrolled in the Temple Infant and Parent Support Services (TIPPS) vs. 437 women (not randomised) receiving usual care (matched for age, parity, ethnicity, health insurance and smoking)	% PTB* (<37 weeks): 4.3% vs. 12.0%, p < 0.005	N/A	Yes/Possibly	N/A
***Tennessee Medicaid Managed Care programme (TennCare)***					
Conover, 2001	Before and after study with an adjacent geographical area as a control group.	*Adjusted Odds Ratio (95% CI)* *for PTB (<37 weeks):*	*Adjusted Odds Ratios (95% CI)* *for neonatal death* *(<28 days):*	*No conclusion* *stated/No*	*No/No*
	IB = Intervention area, 'before' IA = Intervention area, 'after' CB = Comparator area, 'before' CA = Comparator area, 'after' TN = Tennessee NC = North Carolina Sample size (births): IB: 69329 IA:70045 CB: 94012 CA: 94910 Not randomised.	IB vs. CB: 0.764 (0.74-0.79) IA vs. CA: 0.796 (0.77-0.82) Ratio (IA vs. CA)/(CB vs. CA): 1.042 (1.00-1.09)	IB vs. CB: 0.862 (0.74-1.00) IA vs. CA: 1.012 (0.87-1.18) Ratio (IA vs. CA)/ (IB vs. CB): 1.174 (0.95-1.46) *Adjusted Odds Ratios (95% CI)* *for infant death* *(<1 year):* IB vs. CB (TN vs. NC, 'before'): 0.990 (0.88-1.11) IA vs. CA (TN vs. NC, 'after'): 1.146 (1.02-1.29) Ratio(IA vs. CA)/(IB vs. CB): 1.158 (0.98-1.37)		
**b) Programmes providing enhanced antenatal care to socioeconomically disadvantaged women with additional clinical risk factors for PTB/LBW**					
					
***West Los Angeles Preterm Prevention Project***					
Hobel, 1994	1774 high-risk women attending a clinic randomised to provide the PTB prevention programme vs. 880 high-risk women attending a clinic randomised to usual care (clinics unaware of women's risk scores).	*Unadjusted % PTB (<37 weeks):* 7.4% vs. 9.1% (C1), p = 0.063. Adjusted*Odds Ratio(95% CI) *for PTB(<37 weeks):* 0.78 (0.58-1.04). One-sided test for treatment effect: p = .045. * Adjusted for number of high risk problems.	N/A	Yes/Possibly	N/A
***Alabama augmented antenatal care programme for high risk women***					
Klerman, 2001	318 women randomised to receive augmented care vs. 301 women randomised to usual care	*Unadjusted % PTB (undefined):* 10.6% vs. 14.0%, p = 0.22	*N/A*	No/No	N/A
**c) Programmes targeting other vulnerable/at risk groups**					

***New York Prenatal Care Assistance Program (PCAP)***					
Newschaffer, 1998	240 eligible women (HIV infected, drug abusing) who received antenatal care at a *PCAP* participating clinic vs. 113 eligible women who received antenatal care at a non PCAP- participating clinic. Not randomised	*Unadjusted % PTB (<37 weeks):* 13% vs. 22.6%, p = .001 Adjusted* Odds Ratio (95% CI) *for PTB* (<37 weeks): 0.57 (0.34-0.97) *Adjusted for maternal characteristics.	*N/A*	Yes/Possibly	N/A
Turner, 2000	1298 eligible women (HIV infected) who received antenatal care from a PCAP- participating clinic vs. 425 eligible women who received antenatal care from a non PCAP- participating clinic. Not randomised	*Adjusted Odds Ratio (95% CI)* *for PTB (<37 weeks):* 0.53 (0.40-0.70)* *Adjusted for maternal characteristics Additional adjustment for health care and social service use during pregnancy, illicit drug use, and for adequacy of antenatal care attenuates the effect, but effects remain statistically significant.	*N/A*	Yes/Possibly	N/A

#### a) Programmes targeting socioeconomically disadvantaged women without specific clinical risk factors for PTB/LBW

Two linked studies reported by Ickovics [49,50] evaluated the group antenatal care model in disadvantaged populations: the first an observational study conducted in clinics serving low-income, predominantly minority women in Atlanta, Georgia and New Haven, and the second a larger RCT conducted at university-affiliated hospitals in Connecticut and Georgia. The initial evaluation was inconclusive, largely because of the potential risk of selection bias. The subsequent trial reported a significant reduction in PTB in the group care arm (adjusted odds ratio 0.67, 95% confidence interval (CI) 0.44-0.98).

An observational evaluation of the Temple Infant and Parent Support Services (TIPPS) programme [54], a 'customised' comprehensive multidisciplinary service designed to meet the specific needs of the local population in North Philadelphia, Pennsylvania, reported a statistically significant effect on PTB (4.3% vs. 12% preterm in those not enrolled in TIPPS). Because of the risk of selection bias the reviewers considered the findings inconclusive but consistent with a possible beneficial effect.

One study, a before and after study with a contemporaneous comparison group, evaluated a 'managed care' model of delivering antenatal care (the Tennessee Medicaid Managed Care programme (TennCare)) in one US state (Tennessee) against a standard antenatal care model in an adjacent state (North Carolina) [47]. Outcomes (PTB and infant mortality) in the before and after periods did not show any relative improvement in the intervention area compared with the 'control' area. The study did not provide evidence of a beneficial effect of managed care on either PTB or neonatal mortality although some implementation problems occurred during the evaluation which may have affected the outcome.

#### b) Programmes providing enhanced antenatal care to socioeconomically disadvantaged women with additional clinical risk factors for PTB/LBW

A cluster randomized trial of the *West Los Angeles Preterm Prevention Project *[48], a broad, multi-faceted PTB prevention programme, reported a statistically significant reduction in PTB, based on a *one-sided *test for an intervention effect (7.4% PTB in the intervention clinics vs. 9.1% in the control clinics, p = .063 (two-sided), p = .045 (one-sided); adjusted odds ratio 0.78, *two-sided *95% CI 0.58-1.04). Because the effect was of borderline statistical significance and there were concerns about aspects of the statistical methods (see additional file 3), findings were considered inconclusive by the reviewers but consistent with a possible beneficial effect of the intervention on PTB.

An RCT of an augmented antenatal programme in Alabama [43] reported a non-significant reduction in PTB (10.6% PTB vs. 14%, p = 0.22). Findings were considered inconclusive.

#### c) Programmes targeting other vulnerable/at risk groups

An observational evaluation of the *New York Prenatal Care Assistance Program *(PCAP) in HIV positive women [55] reported a significant effect on PTB (<37 weeks) in HIV positive women attending a PCAP-accredited clinic compared with those who received care in a non PCAP-participating clinic (adjusted odds ratio 0.53, 95% CI 0.40-0.70).

A second overlapping observational evaluation of the same programme in HIV positive substance users [38] reported a significant effect on PTB (<37 weeks) compared with HIV positive substance users who received care in a non-PCAP participating clinic (adjusted odds ratio 0.57, 95% CI 0.34-0.97).

In both cases, the reviewers considered that the evidence was inconclusive due to the risk of selection bias in these non-randomised studies but consistent with a possible beneficial effect of PCAP on PTB in both the populations studied.

### Programmes provided as an adjunct to comprehensive antenatal care

Results of the seven studies of adequate quality which evaluated interventions provided as an adjunct to standard antenatal care are summarised in Table 6.

**Table 6:** Effectiveness of interventions provided as an adjunct to comprehensive antenatal care

Study	*Study groups/sample size*	*Effectiveness*		Evidence of effectiveness: authors' conclusion/reviewer assessment	
		
		*PTB outcome*	*Neonatal/infant mortality * *outcome*	*PTB*	Neonatal/ infant mortality
**a) Interventions aimed at socioeconomically disadvantaged women**					

***Home visiting/telephone support***					
Kafatos, 1991	Florina intervention programme. 296 women attending one of the clinics cluster randomised to provide the interventions vs. 263 women attending one of the clinics randomised to provide normal care.	*Unadjusted % PTB* *(<37 weeks):* 3.7% vs. 8.3%, p < 0.04	*Neonatal deaths, n (%)* *(<27 days):* 6 (2.1%) vs. 5 (2.0%)	Yes/Possibly	No/No
Kitzman, 1997	518 women randomised to receive intensive nurse home-visitation services during pregnancy vs. 681 women randomised to receive normal care during pregnancy.	*Unadjusted % PTB* *(<37 weeks)*: 11% vs. 13% *Unadjusted %* *spontaneous PTB* *(<37 weeks)*: 8% vs. 9% *Adjusted Odds Ratio* *(95% CI) for PTB* *(<37 weeks)*: 0.8 (0.6-1.2) *Adjusted Odds Ratio* *(95% CI) for* *spontaneous PTB* *(<37 weeks)*: 0.8 (0.5-1.3)	*N/A*	*No/No*	*N/A*
***Maternity care coordination***					
Buescher, 1991	15,526 women who received maternity care coordination vs. 34,463 women who did not receive maternity care coordination. Not randomised	N/A	*Unadjusted infant deaths* *per 1000 live births:* 9.9 vs. 12.2, p = 0.02 *Adjusted Odds Ratio* *(95% CI) for* *infant death:* 1.20 (0.98-1.47)	N/A	Possibly/ Possibly
**b) Interventions aimed at or evaluated in socioeconomically disadvantaged women with additional risk factors for PTB/LBW**					

***Home visits/telephone support***					
Bryce, 1991	981 women randomised to receive additional antenatal social support vs. 986 women randomised to receive standard antenatal care.	*Stratified Odds Ratio* *(95% CI) for PTB* *(stratified by* *social class)* 0.84 (0.65-1.09) *Odds Ratios by* *social class*: Professional: 0.59 (0.36-0.96) Clerical: 1.00 (0.64-1.56) Manual: 0.96 (0.59-1.56)	*Neonatal deaths before* *hospital discharge:* 1.4% vs. 0.6% *Postneonatal deaths* *before hospital* *discharge*: 0% vs. 0.2%	No/No	No conclusion stated/No
Moore, 1998	775 women randomised to receive the nurse telephone intervention vs. 779 women randomised to receive usual care.	*% PTB (<37 weeks)* 9.7% vs. 11.0%; Relative Risk (RR) (95% CI): 0.87 (0.62-1.22), p = 0.415 Stratified analysis: Black women, aged < = 18 years: 11.0% vs. 7.9% RR: 1.39 (0.72,2.67), p = 0.039 Black women, aged > = 19 years: 8.7% vs. 15.4% RR: 0.56 (0.38-0.84), p = 0.004 White or other women, aged < = 18 years: 7.8% vs. 4.1% RR: 1.92 (0.61-6.02), p = 0.255 White or other women, aged > = 19 years: 19.6% vs. 6.6% RR: 2.99; (0.98-9.09), p = 0.041	*N/A*	No*/No *Authors conclude intervention effective in subgroup of black women aged ≥19	N/A
Oakley 1990	255 women randomised to receive social support plus usual care vs. 254 women randomised to receive usual care	*% PTB (<37 weeks):* 18% vs. 19% % by gestational age: <28 weeks: 2% vs. 1% 28-32 weeks: 3% vs. 4% 33-36 weeks: 13% vs. 14% 37+ weeks: 82% vs. 81%	*Neonatal deaths (%)*: 1% vs. 1%	No conclusion stated/No	No conclusion stated/No
**c) Interventions evaluated in other vulnerable/at risk groups**					

***Higgins Nutrition Intervention Program***					
Dubois, 1997	1203 adolescents who participated in the Higgins Nutrition Intervention during pregnancy vs. 1203 adolescents (matched on site, year and age) who did not receive the intervention. Not randomized.	Unadjusted % PTB (<37 weeks): 8.2% vs. 12.8% *Unadjusted %* *very preterm* *(<34 weeks)*: 2.3% vs. 5.1% Adjusted Odds Ratio (95% CI) for PTB (<37 weeks): 0.59 (0.45 - 0.78), p < = 0.001 *Adjusted Odds Ratio* *(95% CI)* *for very preterm birth* *(<34 weeks)* 0.53 (0.35 - 0.81), p < = 0.001 Odds ratios also reported for subsamples-pregravid weight <50 kg; pregravid weight 50 kg or more; 13-17 yrs; 18-19 yrs.	*N/A*	Yes/Possibly	N/A

#### a) Interventions aimed at socioeconomically disadvantaged women

Three studies evaluated programmes aimed at socioeconomically disadvantaged women in general: two evaluated home visiting programmes and one evaluated maternity care coordination.

A cluster RCT evaluating the antenatal component of a home visiting programme with a focus on nutritional education, delivered to an isolated rural population (Florina) in Northern Greece [42], reported a significant effect on PTB (3.7% PTB in the intervention group vs. 8.3% in the comparator group, p < 0.04). Because the effect was of borderline statistical significance and there were concerns about aspects of the statistical methods (see additional file 3), findings were considered inconclusive but consistent with a possible beneficial effect of the intervention on PTB.

A well-designed RCT to evaluate the antenatal home visiting component of the *Prenatal and Early Childhood Nurse Home Visitation Program *in multi-disadvantaged, black, low-income women in Tennessee [51], found no evidence of a beneficial effect on PTB (11% PTB in the intervention group vs. 13% in the comparator group; adjusted odds ratio 0.8 (95% CI 0.6-1.2)).

A large retrospective observational evaluation of a maternity care coordination programme provided to Medicaid recipients in North Carolina [23] reported a statistically significant effect on infant mortality (adjusted odds ratio 1.20, 95% CI 1.47-0.98). Because of the risk of residual confounding, the reviewers considered the findings inconclusive but consistent with a possible beneficial effect of the intervention on infant mortality.

#### b) Interventions aimed at or evaluated in socioeconomically disadvantaged women with additional risk factors for PTB/LBW

Three studies evaluated home visiting/telephone support programmes provided to women with additional risk factors for PTB/LBW.

An RCT of antenatal support delivered through home visits and telephone calls to women with a prior PTB or other risk factors for PTB in Western Australia [46] did not demonstrate a significant beneficial effect on PTB in a socioeconomically mixed population of higher risk women (odds ratio 0.84; 95% CI 0.65-1.09); a stratified analysis by social class suggested that the beneficial effect, if any, was confined to the most advantaged women in the study. Odds ratios for women classified as 'clerical' and 'manual' were close to one.

An RCT of an intervention involving telephone assessment/advice in North Carolina [52] also found no significant beneficial effect on PTB overall but reported a beneficial effect in a subgroup of black women aged > = 19 years (relative risk 0.56, 95% CI 0.38-0.84, p = 0.004). It is unclear if the sub-group analysis by age and ethnicity was pre-specified. The study was not considered to provide evidence of a beneficial effect overall; the subgroup analysis was considered inconclusive but consistent with a possible beneficial effect in black women aged > = 19.

An RCT of a nurse home visiting programme in the UK, aimed at socioeconomically disadvantaged women with a prior LBW birth [53], similarly found no effect on PTB (18% PTB in the intervention group vs. 19% in the usual care arm; odds ratio not reported).

#### c) Interventions evaluated in other vulnerable/at risk groups

An observational evaluation of a nutritional programme, the *Higgins Nutrition Intervention Program*, in adolescents [41] reported a substantial, statistically significant effect on PTB (<37 weeks) (adjusted odds ratio 0.59, 95% CI 0.45-0.78) and on early PTB (<34 weeks) (adjusted odds ratio 0.53, 95% CI 0.35-0.81). Although the study was inconclusive due to the risk of selection bias, the reviewers considered the findings consistent with a possible beneficial effect on PTB.

## Discussion

The purpose of this review was to evaluate the effectiveness of interventions focused on the delivery or organisation of antenatal care as a means of reducing infant mortality or its three major causes (PTB, congenital anomalies, SIDS/SUDI) in disadvantaged and vulnerable women.

We identified 36 primary reports of eligible studies evaluating interventions in a range of disadvantaged and vulnerable populations including socioeconomically disadvantaged/low-income women in general, socioeconomically disadvantaged/low-income women with additional clinical risk factors for adverse pregnancy outcome, and four other specific groups at risk of adverse pregnancy outcome: teenagers, substance users, indigenous women and HIV positive women.

Overall, the quality of evidence was poor and, for most of the interventions considered, there was insufficient evidence to evaluate consistency of findings across multiple studies. Less than half of the included evaluations were considered to have 'adequate' internal validity. Even for interventions shown to be effective in higher quality studies, such as group antenatal care, we considered that the evidence was too sparse to reliably conclude that the interventions were effective in reducing PTB or neonatal mortality in the disadvantaged and vulnerable populations considered, or that the findings could be generalised to other disadvantaged populations.

We concluded that the evidence relating to seven interventions, although inconclusive, indicated a possible beneficial effect on PTB or on infant mortality.

The following four models of comprehensive antenatal care were considered promising:

• Findings of one well-conducted RCT [49] suggested that group antenatal care might reduce PTB in socioeconomically disadvantaged women. A cohort study evaluating the same model of group antenatal care [50] did not show a consistent beneficial effect on PTB, but the study was too small to detect an effect on this outcome. The group antenatal care model is well defined and described and would appear to be transferable to non-US healthcare systems.

• Trials of two broad, multifaceted, clinic-based PTB prevention programmes targeting disadvantaged women with additional clinical risk factors for PTB suggested that such interventions might be effective in reducing PTB. The two interventions evaluated [43,48] were not identical but appeared to share the common approach of targeting a broad range of risk factors in women identified as being at higher-risk. Such programmes would potentially be transferable to non-US healthcare systems, although only one of the two reports provided sufficient detail to enable replication of the main elements of the programme [43].

• The intensive, multi-component TIPPS programme evaluated by Reece [54] was considered promising with regard to possible effects on PTB despite methodological limitations of the evaluation. The TIPPS intervention itself was designed specifically to address the problems and needs of a disadvantaged local population in North Philadelphia and it is unclear whether the intervention is transferable or the findings generaliseable to other setting. However, some elements of the intervention and the need-based approach to developing 'locally customised' services may merit further examination and evaluation.

• The two overlapping evaluations of the New York *Prenatal Care Assistance Program *(PCAP) [38,55] suggested that the PCAP programme might be effective in reducing PTB in HIV positive women, some of whom were drug users. The programme aims to improve outcomes by improving the quality of care through a process of clinic accreditation with financial incentives to 'accredited' providers. The effect of PCAP on other outcomes has also been evaluated in a wider population of socioeconomically disadvantaged women [56]. The use of enhanced payments to providers providing enhanced services is potentially transferable to other healthcare systems but it is unclear whether the specific services covered by PCAP accreditation would be relevant in other settings.

Three interventions provided as an adjunct to standard antenatal care were also considered promising:

• Two nutritional programmes were tentatively considered promising. An evaluation of the *Higgins Nutrition Intervention Program *in pregnant teenagers indicated a possible beneficial effect on PTB in this population, despite the methodological limitations of the study [41]; and the evaluation of a home visiting programme focussing on nutritional education (the *Florina Intervention Program*) also suggested a possible beneficial effect on PTB in a low-income rural population in Greece [42,57]. The *Florina Intervention Program *was evaluated in isolated agricultural population in Greece with a low-calorie, seasonal diet based on home produce and domestic livestock [57]; the relevance and generalisability of the nutritional elements of the intervention to more urbanised populations is unclear.

• A single US-based study indicated that maternity care coordination might have a beneficial effect on infant mortality in socially disadvantaged women in the USA [23]. However, it is unclear to what extent these findings can be generalised to other healthcare systems since some elements of the intervention may be specific to the healthcare and welfare systems in the USA.

Although we identified seven studies evaluating 'teen' clinics, no conclusions could be drawn regarding the effectiveness of such clinics because of problems of study design and selection bias in the included studies.

We found insufficient evidence of adequate quality to draw any conclusions regarding the effectiveness of the other interventions evaluated.

### Strengths and limitations of this systematic review

In line with our aim to identify the best available evidence on antenatal care interventions targeting socially disadvantaged and vulnerable women we did not restrict ourselves to particular study designs and we designed our searches to reflect this breadth of interest. This lack of specificity may be seen as both strength and a weakness of this review.

The inclusion of less methodologically rigorous evaluations increased the volume of material identified and reviewed and also presented methodological challenges with regard to quality assessment. Furthermore, in practice, it did not add greatly to the evidence regarding effectiveness. Nevertheless, the inclusion and systematic quality appraisal of such evaluations may have served the useful function of highlighting the lack of robust evidence supporting the effectiveness of some widely studied interventions, e.g. 'teen' clinics.

The decision to review a broad category of interventions-antenatal care programmes involving the delivery or organisation of antenatal care-rather than identifying specific interventions *a priori*, has enabled us to provide an overview of a wide range of interventions. A more focussed approach examining a smaller range of specific interventions would have been more consistent with standard systematic reviewing methods, although developing and applying precise interventions definitions-required to ensure reproducible selection of studies-would potentially have been challenging. Furthermore, such an approach would have lacked the flexibility to review a broad, rather diffuse and poorly defined evidence base which was possible with our more comprehensive approach. However, a disadvantage is that a more comprehensive approach necessitates a degree of *post hoc *decision making [32]. For example, following our initial searches we had to decide how best to classify and group the interventions. It is possible that different ways of classifying and grouping the interventions might have changed the 'weight of evidence' in favour of an intervention within scope of the review, but, given the limitations of the evidence, we think it unlikely that this would have resulted in major changes to our conclusions.

An unanticipated consequence of our 'generic' inclusion/exclusion criteria was the exclusion of some seemingly relevant interventions provided as an 'add on' to normal antenatal care. For example, studies relating to some welfare-based US programmes (such as the Special Supplemental Food Program for Women, Infants and Children (WIC)) were excluded not because the intervention was ineligible but because studies evaluating the intervention typically compared 'intervention recipients' with 'non-recipients', with the latter group including women who received no antenatal care. The studies were therefore excluded because they lacked a comparator group receiving standard antenatal care.

It is possible that we may have missed some relevant 'add on' interventions as a result of using non-specific antenatal care search terms (e.g. 'prenatal care') instead of more intervention specific terms. Similarly, socioeconomically disadvantaged study populations are not consistently indexed or mentioned in searchable elements of the bibliographic record. We took some additional steps to increase ascertainment of relevant material, including using an adapted version of an 'equity filter' (developed by the EPPI-Centre to identify material relating to health inequalities) in our searches, and 'snowballing' [58].

Although the titles of articles lacking an abstract were screened and the full-text retrieved where appropriate, there is the possibility that relevant studies lacking an abstract may have been missed; non-English language articles lacking an English abstract were not included.

### Findings in relation to other published evidence

One previous review conducted in the early 1990 s sought to evaluate the "best" evidence relating to the effect of antenatal healthcare programmes on pregnancy outcomes, including infant mortality and gestational age at birth [21]. The authors concluded that maternal care coordination, home visits by nurses and specially targeted smoking and nutritional programmes were associated with "optimized pregnancy outcomes for certain groups of women, including the poor and very young." Nevertheless, as in the present review, and for similar reasons, they urged caution in applying these findings.

Other published reviews have addressed the effectiveness of a range of specific antenatal care interventions but most without a focus on effectiveness in disadvantaged or vulnerable groups of pregnant women:

• **PTB prevention educational programmes for high risk women **A systematic review and meta-analysis of RCTs of PTB prevention educational programmes [59] concluded that they appeared to have little benefit in reducing PTB and might result in an increased rate of diagnosis of preterm labour.

• **Home visiting programmes **A review of the effect of home visits on a range of pregnancy outcomes including PTB (<37 weeks) [26] found that home visiting programmes in general, and more specific programmes (those providing social support and those providing medical care to women with complications) did not improve the preterm delivery rate or other pregnancy outcomes. A second review of interventions involving support during pregnancy for women at increased risk of LBW babies [60], found no effect on PTB (Risk ratio 0.92, 95% CI 0.83 - 1.01). A further 'review of reviews' [61] similarly concluded that there was insufficient evidence to suggest that home-visiting programmes had a beneficial impact on low birth weight or other pregnancy outcomes.

• **Telephone support **A recent review of telephone support interventions concluded that they were ineffective at reducing PTB [25].

• **Nutritional interventions **A review of the effectiveness of interventions to optimize gestational weight gain and diet in pregnant adolescents [62] concluded that such interventions had achieved "promising results" with regard to a range of pregnancy outcomes but found little evidence relating to effects on PTB. The review did not systematically assess the quality of the included material but noted that much of the evidence was methodologically flawed. A further review assessed the effects of a range of nutritional interventions during pregnancy, including advice to increase or reduce energy or protein intake [63]. The authors concluded that although dietary advice appeared to be effective in increasing pregnant women's energy and protein intakes it was unlikely to confer major benefits on infant or maternal health. These findings do not support our tentative conclusions regarding the potentially 'promising' effect of the two programmes with a nutritional focus included in the present review (the Higgins nutritional intervention in teenagers [41], and the Florina home visiting programme which has a nutritional counselling focus [42]) and, on balance, may suggest that a more cautious interpretation of the evidence in favour of these two interventions would be warranted.

• **Midwife-led antenatal care **A Cochrane review [64] did not find a significant beneficial effect of midwife-led antenatal care on PTB compared with other models of care (risk ratio 0.87, 95% CI 0.73-1.04). A second overlapping review of continuity of midwifery care vs. standard care [28] additionally found no significant effect on neonatal mortality (odds ratio 1.27, 95% CI 0.49 - 3.34). A third review examined the evidence relating to various aspects of antenatal care for low-risk women including the effectiveness of midwife/general practitioner-managed care vs. obstetrician/gynaecologist-led shared care [30] also found no significant effect on PTB (relative risk 0.80, 95% CI 0.59 - 1.10).

• **Antenatal care targeting specific vulnerable groups **Rumbold and Cunningham reviewed the impact of antenatal care interventions on Australian indigenous women [24]. They did not assess the quality of the included studies so the interpretation of their findings is uncertain.

With the exception of the findings relating to the possible ineffectiveness of nutritional interventions noted above, the findings of other published reviews appear consistent with our assessment of the effectiveness of antenatal care programmes in disadvantaged and vulnerable populations.

## Conclusions

In summary, we found insufficient evidence of adequate quality to conclude that interventions involving alternative models of organising or delivering antenatal care have been demonstrated to be effective in reducing infant mortality or PTB in socially disadvantaged or vulnerable populations compared with standard models of antenatal care. A small number of the interventions reviewed here were considered 'promising' in terms of their effect on PTB in socially disadvantaged or vulnerable populations, but the effects, if any, are likely to be modest and further robust evaluation would be required before routine adoption of these interventions could be recommended.

## List of abbreviations

OECD: Organisation for Economic Co-operation and Development; WIC: Special Supplemental Nutrition Program for Women, Infants and Children

## Competing interests

The authors declare that they have no competing interests.

## Authors' contributions

JH, JK, PB and RG were involved in study concept and design; JH and RG developed the search strategy; JH ran the searches, carried out screening, quality appraisal and data extraction with the assistance of LO and other members of the review team (see acknowledgements); JK and RG assisted with full-text screening and quality appraisal in cases of disagreement between reviewers; JH wrote the study report and drafted the manuscript; all authors were involved in review and approval of the final manuscript.

## Pre-publication history

The pre-publication history for this paper can be accessed here:

http://www.biomedcentral.com/1471-2393/11/13/prepub

## Supplementary Material

Additional file 1 **Details of the search strategies used in the review**. Click here for file

Additional file 2 **Reasons for exclusion during screening**. Click here for file

Additional file 3 **Study quality: results of GATE assessment**. Click here for file

Additional file 4 **Overview of the intervention characteristics, by target population**. Click here for file
