# Supplementary material for: The effectiveness of antenatal care programmes to reduce infant mortality and preterm birth in socially disadvantaged and vulnerable women in high-income countries: a systematic review
Source: BMC Pregnancy Childbirth. 2011 Feb 11;11:13. doi: 10.1186/1471-2393-11-13 (PMC3050773; doi:10.1186/1471-2393-11-13)
Supplement: Additional file 1 — Details of the search strategies used in the review. [file 1471-2393-11-13-S1.PDF]

## Additional file 1 – Details of the search strategies used in the review

- Section 1 provides details of the main bibliographic database searches
- Section 2 lists the additional online resources searched.

### 1. Bibliographic database searches

Medline, Embase, PsycINFO and HMIC were searched using the Ovid SP interface; Cinahl, was searched using the EBSCO interface; Central and DARE were searched via the Cochrane Library; MIDIRS was searched by the MIDIRS librarian using a keyword search adapted from the MEDLINE search strategy. All searches were run in mid-August 2008

We applied limits and filters to restrict the searches to articles:

- Published from 1990 onwards
- Relating to human subjects
- In English (with or without an abstract) or non-English with an English language abstract

### Medline search strategy

| | **Outcome terms** |
|---|---|
| 1 | exp Infant Mortality/ |
| 2 | exp Perinatal Mortality/ |
| 3 | ((infant$ or perinat$ or neonat$ or postneonat$) adj2 (death$ or mortalit$ or surviv$)).ti,ab. |
| 4 | ((newborn$ or infant$ or perinat$ or neonat$ or postneonat$) adj2 (death$ or dead or died or mortalit$ or surviv$)).ti,ab. |
| 5 | or/1-4 |
| 6 | exp Infant, Premature/ |
| 7 | exp obstetric labor, premature/ or exp premature birth/ |
| 8 | ((preterm or prematur$) adj2 (labour$ or labor$ or birth$ or deliver$ or infant$)).ti,ab. |
| 9 | (prematurity or preterm).ti,ab. |
| 10 | or/6-9 |
| 11 | exp Sudden Infant Death/ |
| 12 | "sudden unexpected death in infancy".ti,ab. |
| 13 | "sudden unexplained death in infancy".ti,ab. |
| 14 | cot death$.ti,ab. |
| 15 | crib death$.ti,ab. |
| 16 | (SIDS or SUDI).ti,ab. |
| 17 | "sudden infant death syndrome".ti,ab. |
| 18 | or/11-17 |

19 exp Congenital Abnormalities/

20 ((birth or congenital) adj2 (defect$ or deform$ or abnorm$ or anomal$ or malform$)).ti,ab.

21 or/19-20

22 5 or 10 or 18 or 21

**Intervention terms**

23 exp Prenatal Care/ or maternal health services/

24 exp Midwifery/

25 ((antenatal or prenatal) adj2 (care or clinic or program* or service*)).ti,ab.

26 or/23-25

**Disadvantaged and vulnerable group terms**

27 exp Socioeconomic Factors/ or exp Social Class/

28 (equity or inequalit$ or equalit$ or unequal$ or inequit$ or disparit$ or gap or gaps or gradient$ or disadvantag$ or socioeconomic$).ti,ab.

29 health inequalit$.mp. or Health Status Indicators/ or *Health Status Disparities/ or *Healthcare Disparities/

30 exp Poverty/ or exp Medical Indigency/ or vulnerable populations/

31 exp Minority Health/ or exp Minority Groups/ or population groups/ or exp ethnic groups/ or health services, indigenous/

32 (ethnic or (black adj2 asian)).ti,ab.

33 (multiethnic$ or multi ethnic$ or multiracial$ or multi racial$).ti,ab.

34 exp Prisoners/ or prison*.ti,ab.

35 exp refugees/ or "Emigrants and Immigrants"/ or "Transients and Migrants"/

36 (immigrant* or refugee* or migrant* or asylum seeker*).ti,ab.

37 exp gypsies/ or travel?er*.ti,ab.

38 exp Homeless Youth/ or exp Homeless Persons/ or homeless$.ti,ab.

39 exp Spouse Abuse/ or Domestic Violence/ or exp battered women/

40 ((abuse$ or violen$) adj4 (partner$ or wife or wives or spouse$ or domestic)).ti,ab.

41 ((neighbo?rhood or economic or rural or urban) adj2 (depriv$ or poverty)).ti,ab.

42 (disadvantag* or deprived area* or innercit* or inner cit*).ti,ab.

43 Mental Disorders/ or exp eating disorders/ or exp mood disorders/ or exp "schizophrenia and disorders with psychotic features"/

44 ((mental$ or psych$) adj2 (ill$ or disorder$ or impair$ or disturb$ or disabil$)).ti,ab.

45 Learning Disorders/ or Mental Deficiency/

46 ((mental$ or learning or cognitiv$) adj2 (retard$ or handicap$ or disab$ or difficult$ or impair$)).ti,ab.

47 exp Prostitution/ or sex worker*.ti,ab.

48 Adolescent Health Services/ or exp Adolescent/ or exp Pregnancy in Adolescence/

49 (teen$ or youth$ or adolescen$).ti,ab.

50 (late adj2 (book$ or initiat$ or attend$)).ti,ab.

51 exp Obesity/ or exp Obesity, Morbid/

52 (obese or obesity).ti,ab.

53 exp HIV Infections/ or HIV/

54 (HIV or HIV-pos$ or HIV-inf$).ti,ab.

55 exp Street Drugs/ or exp Narcotics/ or exp Cocaine/ or exp Crack Cocaine/ or exp Heroin/ or exp amphetamines/ or exp methadone/

56 exp substance-related disorders/ or exp Substance Abuse, Intravenous/ or exp amphetamine-related disorders/ or exp cocaine-related disorders/ or exp marijuana abuse/ or exp opioid-related disorders/ or exp heroin dependence/ or exp phencyclidine abuse/ or exp psychoses, substance-induced/ or exp substance abuse, intravenous/ or substance withdrawal syndrome/

57 exp alcohol-related disorders/ or exp alcoholism/ or exp alcohol-induced disorders/

58 or/27-57

59 22 and 26 and 58

**Limits**

60 limit 59 to (humans and yr="1990 - 2008")

61 limit 60 to abstracts

62 limit 60 to english language

63 61 or 62

64 Case Reports/

65 63 not 64

EMBASE search strategy

1 exp Infant Mortality/ or exp Perinatal Mortality/ or exp Newborn Mortality/ or exp Perinatal Death/
2 ((infant$ or perinat$ or neonat$ or postneonat$) adj2 (death$ or mortalit$ or surviv$)).ti,ab.
3 ((newborn$ or infant$ or perinat$ or neonat$ or postneonat$) adj2 (death$ or dead or died or mortalit$ or surviv$)).ti,ab.
4 or/1-3
5 exp "immature and premature labour"/ or exp immaturity/ or exp premature labor/ or exp prematurity/
6 exp obstetric labor, premature/ or exp premature birth/
7 ((preterm or prematur$) adj2 (labour$ or labor$ or birth$ or deliver$ or infant$)).ti,ab.
8 (prematurity or preterm).ti,ab.
9 or/5-8
10 exp Sudden Infant Death Syndrome/
11 "sudden unexpected death in infancy".ti,ab.
12 "sudden unexplained death in infancy".ti,ab.
13 cot death$.ti,ab.
14 crib death$.ti,ab.

15 (SIDS or SUDI).ti,ab.
16 "sudden infant death syndrome".ti,ab.
17 or/10-16
18 exp Congenital Malformation/
19 ((birth or congenital) adj2 (defect$ or deform$ or abnorm$ or anomal$ or malform$)).ti,ab.
20 or/18-19
21 4 or 9 or 17 or 20
22 exp prenatal care/ or exp maternal care/
23 ((antenatal or prenatal) adj2 (care or clinic or program* or service*)).ti,ab.
24 exp midwife/
25 or/22-24
26 exp social status/ or exp social class/ or exp socioeconomics/
27 (equity or inequalit$ or equalit$ or unequal$ or inequit$ or disparit$ or gap or gaps or gradient$ or disadvantag$ or socioeconomic$).ti,ab.
28 health inequalit$.mp.
29 vulnerable population/ or exp indigent/ or exp poverty/ or exp lowest income group/ or exp medically underserved/
30 exp minority group/ or exp "ethnic and racial groups"/ or exp ethnic group/
31 (ethnic or (black adj2 asian)).ti,ab.
32 (multiethnic$ or multi ethnic$ or multiracial$ or multi racial$).ti,ab.
33 exp Prisoners/ or prison*.ti,ab.
34 exp immigrant/ or exp refugee/ or exp migration/ or exp illegal immigrant/
35 (immigrant* or refugee* or migrant* or asylum seeker*).ti,ab.
36 exp gipsy/ or travel?er*.ti,ab.
37 exp Homelessness/ or homeless$.ti,ab.
38 domestic violence/ or exp battered woman/ or exp partner violence/
39 ((abuse$ or violen$) adj4 (partner$ or wife or wives or spouse$ or domestic)).ti,ab.
40 ((neighbo?rhood or economic or rural or urban) adj2 (depriv$ or poverty)).ti,ab.
41 (disadvantag* or deprived area* or innercit* or inner cit*).ti,ab. )
42 Mental Disease/ or exp eating disorder/ or exp mood disorders/ or exp psychosis/
43 ((mental$ or psych$) adj2 (ill$ or disorder$ or impair$ or disturb$ or disabil$)).ti,ab.
44 Learning Disorder/
45 ((mental$ or learning or cognitiv$) adj2 (retard$ or handicap$ or disab$ or difficult$ or impair$)).ti,ab.
46 exp prostitution/ or sex worker*.ti,ab.
47 exp adolescent pregnancy/ or exp adolescent/
48 (teen$ or youth$ or adolescen$).ti,ab.
49 (late adj2 (book$ or initiat$ or attend$)).ti,ab.
50 exp Obesity/ or exp Morbid Obesity/
51 (obese or obesity).ti,ab.
52 exp Human Immunodeficiency Virus/ or exp Human Immunodeficiency Virus Infection/
53 (HIV or HIV-pos$ or HIV-inf$).ti,ab.
54 exp substance abuse/ or exp drug abuse/ or exp Street Drug/ or exp Illicit Drug/ or exp Narcotic Dependence/ or exp cocaine dependence/ or drug dependence/ or exp heroin dependence/ or exp opiate addiction/
55 exp cocaine/ or exp Cannabis/ or exp Methadone/ or exp heroin/ or exp methamphetamine/ or exp Phencyclidine/
56 exp Withdrawal Syndrome/ or exp addiction/

57 exp Alcoholism/ or exp alcohol abuse/
58 or/26-57
59 21 and 25 and 58
60 limit 59 to (human and yr="1990 - 2008")
61 limit 60 to abstracts
62 limit 60 to english language

**2. Other databases and online resources**

The following specialist databases and online resources were searches to identify potentially eligible primary reports and/or guidelines, reviews and reports which might contain relevant citations:

- Cochrane Database of Systematic Reviews
- Health Technology Assessment Database
- NHS Economic Evaluations Database
- System for information on Grey Literature in Europe (OpenSigle)
- National Guideline Clearing House
- National Institute for Health and Clinical Excellence (NICE)
- National Library for Health
- Health Development Agency National Institute for Health Research Service Delivery and Organisation Programme (SDO)
- Social Care Online
- Research Register for Social Care

The Cochrane Database of Systematic Reviews, the Health Technology Assessment Database and the NHS Economic Evaluations Database were all searched via the Cochrane Library advanced search facility using the Medline search strategy; all other online databases were searched using relevant keywords such as: antenatal care, prenatal care, maternity care, pregnancy, socioeconomic, vulnerable, socially disadvantaged, ethnicity, teenagers, adolescents.
