# Supplementary material for: The effectiveness of antenatal care programmes to reduce infant mortality and preterm birth in socially disadvantaged and vulnerable women in high-income countries: a systematic review
Source: BMC Pregnancy Childbirth. 2011 Feb 11;11:13. doi: 10.1186/1471-2393-11-13 (PMC3050773; doi:10.1186/1471-2393-11-13)
Supplement: Additional file 2 — Reasons for exclusion during screening. [file 1471-2393-11-13-S2.PDF]

Additional file 2 - Reasons for exclusion during screening

	Number excluded<br>Screened on:	
	Title/abstract	Full-text
<u>General</u>		
Not primary research	997	5
Not a journal article	3	
<u>Population</u>		
Not OECD	758	1
Not relevant population (pregnant women)	153	
<u>Intervention</u>		
No intervention	1345	
Not antenatal care intervention	2	4
Management of labour/birth	13	2
Standard antenatal care only		2
Ineligible clinical intervention		15
Peri-conceptional intervention		6
Methadone or opiate substitution		3
Stand alone intervention - not delivered/evaluated in conjunction with antenatal care or unclear if delivered/evaluated in conjunction with antenatal care		18
<u>Comparator</u>		
No comparator/control group	106	6
Care in comparator group not standard antenatal care		1
<u>Outcome</u>		
No relevant outcome	126	15
Outcome not reported in relevant population		16
<u>Other</u>		
Not an effectiveness evaluation	94	3
Study inappropriately designed to address review question (intervention and comparator groups differ; time series lacking clearly defined intervention/comparator groups)		5
Other (unclear if comparator group received antenatal care)		1
TOTAL EXCLUDED	3597	103
