# Supplementary material for: The effectiveness of antenatal care programmes to reduce infant mortality and preterm birth in socially disadvantaged and vulnerable women in high-income countries: a systematic review
Source: BMC Pregnancy Childbirth. 2011 Feb 11;11:13. doi: 10.1186/1471-2393-11-13 (PMC3050773; doi:10.1186/1471-2393-11-13)
Supplement: Additional file 3 — Study quality: results of GATE assessment. [file 1471-2393-11-13-S3.PDF]

Additional file 3 – Study quality: results of GATE assessment

Study	Study design	GATE assessment	Reviewer comments
**a) Randomised Controlled Trials**			
Bryce (1991)	RCT	Mixed	RCT with randomisation before consent. Trial not restricted to socioeconomically disadvantaged women but stratified analysis by social class reported.
Collaborative Group on PTB Prevention (1993)	RCT	Poor	Authors note that results did not show a consistent effect with significant, unexplained heterogeneity between the five study sites. Possible contamination of control group (ie exposure to intervention)
Goldenburg* (1990)	RCT	See primary report	Report from single site of the Collaborative Group on PTB Prevention (1993) study.
Hobel (1994)	Cluster RCT	Mixed	Cluster randomisation of small number of sites did not adequately balance groups wrt PTB risks; not possible to assess likelihood of residual confounding. Analysis did not take account of cluster randomisation so confidence intervals underestimated. Unlear if one-sided test of significance was pre-specified.
Ickovics (2007)	RCT	Mixed	Generally well designed/reported RCT.
Kafatos (1991)	Cluster RCT	Mixed	Details of randomisation process (ie concealment) unclear but groups appeared to be generally well balanced with a marginally higher prevalence of obstetric risk factors present in the intervention group. Analysis did not take account of cluster randomisation so confidence intervals underestimated
Kitzman (1997)	RCT	Good	Well designed/reported RCT.
Klerman (2001)	RCT	Mixed	Generally well designed/reported RCT but underpowered for PTB outcome.
Moore (1998)	RCT	Mixed	Well designed/reported RCT.
Oakley (1990)	RCT	Good	Well designed/reported RCT.

Study	Study design	GATE assessment	Reviewer comments
**b) Observational studies**			
Armstrong (2003)	Retrospective cohort	Poor	Authors' conclusion based on comparison with non-substance abusing comparator
Benussen-Wall (2001)	Retrospective cohort	Poor	Small study; groups differed due to imperfect matching; high risk of confounding.
Bienstock (2001)	Retrospective cohort	Poor	Potential selection bias; managed care compared with a single hospital site
Buescher (1991)	Retrospective cohort	Mixed	Design subject to risk of selection bias but adequate adjustment for a range of confounders and various additional analyses conducted to investigate potential biases.
Burkett (1998)	Prospective cohort	Poor	High risk of selection bias (intervention group compared with those who refused the intervention); no adjustment for potential confounders.
Clark (1993)	Retrospective cohort	Poor	Selection bias. No controlling for confounders. Intervention and comparator groups arguably not drawn from the same target population (differed with respect to income).
Conover (2001)	Other observational	Mixed	Implementation problems occurred during the evaluation period leading to possible failure to fully deliver the intervention.
Das (2007)	Before and after	Poor	Before and after study: high risk of bias due to secular changes in outcome; possible difference in prognostic factors (particularly age) in the intervention and control groups. Underpowered.
Dubois (1997)	Retrospective observational	Mixed	High risk of selection bias but risk profiles of intervention and comparator groups suggest that the intervention group had a higher baseline risk of adverse pregnancy outcome.
Edwards (1995)	Retrospective cohort	Poor	Risk of selection bias and no adjustment for confounding.
Grady (2004)	Mixed prospective/ retrospective cohort	Poor	High risk of selection bias and no control of confounding.
Ickovics (2003)	Prospective cohort	Mixed	Self-selection bias (volunteers). Study powered for birth weight outcome, not neonatal mortality or preterm delivery
Keeton (2004)	Retrospective cohort	Poor	Strong selection bias with high risk of residual confounding despite statistical adjustment for range of confounders

Study	Study design	GATE assessment	Reviewer comments
Lane (2001)	Before and after	Poor	Before and after study: changes in infant mortality cannot be reliably attributed to the study intervention. The comparison with temporal trends in the surrounding area is inconclusive since the socioeconomic and racial mix of the two areas differs.
Lenaway (1998)	Cohort	Poor	High risk of selection bias; impossible to assess comparability of comparator counties; substantial 'baseline differences', no adjustment for confounding.
Mackerras (2001)	Before and after	Poor	Before and after study; the authors note that it is not possible to separate the effects of concurrent changes in the health services in the communities studied from the effects of the intervention
Miles (2007)	Before and after	Poor	Small before and after study.
Morris (1993)	Retrospective cohort	Poor	High risk of selection bias and inadequate adjustment for confounding.
Mvula (1998)	Prospective cohort	Poor	Risk of selection bias; potential for channelling of higher risk patients to hospital clinic not adequately addressed.
Newshaffer* (1998)	Retrospective cohort	Mixed	Secondary report – study population overlaps with that reported in (2000). Large, generally well designed/reported multi-site retrospective observational study. Risk of selection bias but adequate statistical adjustment for range of potential confounders.
Panaretto (2007)	Before and after with additional contemporary comparator group	Poor	Before and after study; high risk of selection bias; increased use of ultrasound during the study period could have led to more accurate dating of gestational age.
Reece (2002)	Prospective cohort	Mixed	High risk of selection bias but the intervention group was recruited by outreach workers from high risk populations and thus might be expected to have a higher risk profile than those entering prenatal care voluntarily.
Sweeney (2000)	Cohort	Poor	Small study with strong selection bias (intervention group compared with women who enrolled in substance abuse treatment postnatally but refused the intervention while pregnant).
Turner (2000)	Retrospective cohort	Mixed	Large, generally well designed/reported multi-site retrospective observational study. Risk of selection bias but adequate statistical adjustment for range of potential confounders.
Ukil (2002)	Retrospective cohort	Poor	Small study; characteristics of study groups not reported; no adjustment for confounding; multiple outcomes compared (high risk of type I error).
Van Winter (1997)	Prospective cohort	Poor	High risk of selection bias: intervention recipients compared with those who refused the intervention.

* Linked secondary report
