# Supplementary material for: The effectiveness of antenatal care programmes to reduce infant mortality and preterm birth in socially disadvantaged and vulnerable women in high-income countries: a systematic review
Source: BMC Pregnancy Childbirth. 2011 Feb 11;11:13. doi: 10.1186/1471-2393-11-13 (PMC3050773; doi:10.1186/1471-2393-11-13)
Supplement: Additional file 4 — Overview of the intervention characteristics, by target population. [file 1471-2393-11-13-S4.PDF]

Additional file 4 – Overview of the intervention characteristics, by target population

<u>**Comprehensive antenatal care programmes**</u>

a) Programmes targeting socioeconomically disadvantaged women without specific clinical risk factors for PTB/LBW

- Group antenatal care [49, 50]
- Comprehensive multidisciplinary service with outreach [54]
- Comprehensive antenatal care including PTB prevention programme [65]
- Nurse/midwife clinic for low risk women [66, 67]
- Managed care [47]

b) Programmes providing enhanced antenatal care to socioeconomically disadvantaged women with additional clinical risk factors for PTB/LBW

- Broad, multifaceted PTB prevention programme [43, 48]
- Programme with focus on patient education regarding signs of preterm labour plus additional visits/pelvic examinations [68]
- Hospital clinic vs. 'managed care' [69]
- Specialist clinic for women at high risk of PTB (twin gestation) [70]

c) Programmes targeting other vulnerable/at risk groups

Adolescents

- 'Teen' clinics [45, 71-76]
- Adolescent group prenatal care (CenteringPregnancy) [77]

Indigenous women

- Culturally sensitive comprehensive antenatal care including community/outreach services [78, 79]

Low-income , HIV positive women

- Comprehensive care in accredited general antenatal clinics providing an enhanced range of services [55]

Substance users

- Comprehensive care in accredited general antenatal clinic providing an enhanced range of services [38]

<u>**Interventions provided as an adjunct to comprehensive antenatal care**</u>

a) Interventions aimed at socioeconomically disadvantaged women

- Case management/care co-ordination[23, 80]
- Nurse home visits [42, 51]
- Multifaceted 'Healthy Start' programme[81]

b) Interventions aimed at or evaluated in socioeconomically disadvantaged women with additional clinical risk factors for PTB/LBW

- Home visits/telephone support [46, 52, 53]

c) Interventions evaluated in other vulnerable/at risk groups

Adolescents

- Stand alone nutritional intervention [41]

Substance users

- Substance abuse programme– women screened and referred from a general antenatal clinic[44, 82, 83]
- Drug rehabilitation programme provided as an adjunct to antenatal care in a dedicated prenatal substance abuse clinic [40]
